# Considering the Developing Entity in an Artificial Womb as a Patient

**DOI:** 10.1111/bioe.70021

**Published:** 2025-07-16

**Authors:** Frédérique Drouin, Alice Cavolo, Vardit Ravitsky, Charles Dupras

**Affiliations:** ^1^ Department of Social and Preventive Medecine University of Montreal Montréal Quebec Canada; ^2^ Institute of Biomedical Ethics and History of Medicine University of Zurich Canton of Zurich Switzerland; ^3^ The Hastings Center Garrison New‐York USA

**Keywords:** artificial womb, beneficence, ectogenesis, ectogestation, moral status, *The fetus as a patient*

## Abstract

Artificial womb (AW) prototypes are currently being developed with the aim of improving the medical care of extremely premature infants. Despite the seemingly imminent reality of partial ectogenesis (i.e., gestation partially outside a human womb), there is persisting debate about the moral status of the fetus transferred in an AW—henceforth, the “developing entity.” For some, AWs are simply another neonatal intensive cares’ technology. Thus, developing entities in AWs should deserve the same protections as newborns in incubators. Others consider that AWs are fundamentally different technologies than incubators. Therefore, they believe that developing entities in AWs are new moral entities. These differences in perception generate disagreement about how developing entities in AWs should be treated and how decisions about them should be made. We argue that developing entities in AWs should be considered patients by transposing Chervenak and McCullough's “The fetus as a patient” proposition to the context of partial ectogenesis. As pregnant persons will have to consent to transfer their fetuses in AWs, and this technology will ultimately present itself as a beneficial medical intervention for viable developing entities in AWs, these latter would be patients, even if they are not legally and morally recognized as person. Thus, the moral obligations of beneficence and non‐maleficence owed by physicians to their patients would apply to entities in AWs, ethically guiding their treatment and decision‐making toward them.

## 1 Introduction

Artificial womb (AW) prototypes are currently being developed and tested on animals with the aim of improving the medical care of extremely premature infants (EPIs) of 22–25 weeks' gestational age, a population with high morbidity and mortality rates [1].

AW technologies usually take the form of a plastic bag containing synthetic amniotic fluid. Oxygen, blood, and nutrition are supplied via the umbilical cord of the extracted fetus transferred in an AW [2] (henceforth, the “developing entity in an AW”).^1^ The developing entity in an AW can be kept alive without the need for mechanical ventilation. This is seen as potentially beneficial for reducing the risks associated with mechanical ventilation, such as interrupted lung development [3], lung damage, and long‐term disability [4]. The AW could, therefore, be a better option than the incubator currently used in neonatal intensive care (NIC) for EPIs. While relevant, the question of the precise grounds on which AW would be offered and how human candidates would be selected in view of the potentially very high financial cost of this technology are beyond the scope of this paper.

The AW is currently considered for its potential to enable *partial ectogenesis* ^2^ (i.e., the development of a human entity partially carried out outside of a human womb). In contrast, *total ectogenesis* refers to the complete development of a human being, from embryo to delivery, entirely outside of the human body. Several authors, including Romanis and Horn [5], Segers [6], and De Bie et al. [4], have pointed out the futuristic, even unrealistic, aspect of total ectogenesis. However, progress with partial ectogenesis is undeniable. In fact, the *Children's Hospital of Philadelphia* (CHOP), where a research team has already carried out over 300 successful partial ectogenesis trials on sheep fetuses [7], has held discussions with the *Food and Drug Administration* (FDA), in 2023, regarding the potential start of the first clinical trials testing AW prototypes on human beings. Other research teams from different countries, such as the Netherlands, Japan, Spain, Singapore [8], and Canada [9], are also currently developing their own AW prototypes.

Despite the seemingly imminent reality of partial ectogenesis, there is persisting debate on how the developing entity in an AW should be considered. Specifically, ambiguities persist regarding its moral status. For the purpose of synthesis, this article concentrates on the moral status of the developing entity in an AW and not on its legal status.^3^

The question of the nature of AWs is at the heart of debates concerning the moral status of the developing entity in an AW. For some authors such as Wozniak and Fernandes, the AW is simply another technology in the line of NIC, and thus the developing entity in an AW deserves the same protections as the newborn in an incubator [3]. Others, such as Romanis, consider that AW is a fundamentally different technology than the incubator, since the former would support a process more akin to the continuation of a human gestation, while an incubator passively supports the infant [10]. Thus, because of the new and unique capacities of AWs, some authors such as Romanis [10], and Kingma and Finn [11], believe that the developing entity in an AW is a new moral entity, which may be called the “gestateling.” These differences in perception generate disagreement about the moral status that should be accorded to the developing entity in an AW and, by extension, how it should be treated and how decisions about it should be made.

Most existing proposals suggest determining the moral status of the developing entity in an AW according to *what it is* (fetus, newborn, new entity). Most articles overlook or only succinctly address the crucial question of how we ought to ethically treat these entities. In this paper, we argue that Chervenak and McCullough's theory of “The fetus as a patient” [12] (TFAAP) is helpful to address these shortcomings. From this point of view, the developing entity in an AW should be considered as a patient regardless of whether it is seen more as a fetus or a newborn. Furthermore, when an entity is considered a patient, moral and deontological obligations defined within regulations apply to it, diminishing the risks of inconsistent treatment.

Below, we discuss the classification of AWs (either incubator extension or fundamentally new technology) and semantic issues surrounding concepts such as “birth” and “newborn,” as applied to the developing entity in an AW. Using Canada as an example, we show how these terms have moral implications, including on a human entity's right to life. To address the moral status of the developing entity in an AW, we propose enacting Chervenak and McCullough's theory of TFAAP as an alternative. We describe the original version of their proposal, that is, to consider the fetus in the human uterus as a patient, under certain conditions. Hence, we look at how Chervenak and McCullough's proposal can be transposed to the developing entity in an AW in the context of partial ectogenesis. We argue that it is the most promising framework to guide the ethical treatment of the developing entity in an AW and the associated decision‐making process.

## 2 The Moral Status of the Developing Entity in an Artificial Womb: Between Fetus, Gestateling, or Newborn

This section focuses on the moral status of the developing entity in an AW. We adopt Warren's definition of an entity with moral status as “an entity towards which moral agents have, or can have, moral obligations [and that…] we may not treat it in just any way we please” [13]. This definition, while introducing a relational aspect, does not subordinate the moral status of an entity to the action or inaction of moral agents. Warren's definition does not exclude the possibility that an entity endowed with moral status may possess intrinsic value. This value could precisely justify the moral obligations of moral agents towards this entity, preventing them from treating it as they please. Whether or not the developing entity in an AW has intrinsic value—this is not the point—we argue that moral status is, at least in part, relational. For an intrinsic value to actualize, it must be recognized by moral agents; it cannot exist as a free electron if no one acts to recognize and protect that value.

The moral status accorded to the developing entity in an AW will guide morally (un)acceptable decisions and actions regarding this entity. As mentioned above, the way in which AW technology is classified (either incubator extension or fundamentally new technology) influences the way in which the developing entity in an AW is morally considered and treated. For instance, Kingma and Finn believe that AWs are not a continuation of the technologies currently used in NIC, such as the incubator [11]. For them, what differentiates the AW from the incubator is the fact that the former supports the development of entities that retain their fetal characteristics (umbilical cord, oxygenation via the placenta, fetal‐specific cardiac, vascular, and pulmonary structures, etc.). The incubator, on the other hand, is used for newborns that have already acquired neonatal physiological characteristics. Considering that the AW allows the entity inside to retain its fetal characteristics, Kingma and Finn do not consider it a neonate (or newborn), but instead a “gestateling.” They explain: “very roughly, to be a fetus is to have a physiology characteristic of a fetus; and to be a neonate is to have a physiology characteristic of a neonate. To be a gestateling, then, is to have a physiology characteristic of a fetus, but to exist outside of a gestating mammal” [11, p. 359]. Their position aligns with Romanis’ view that birth should be seen as both a change in the developing environment *and* in the physiological characteristics of the developing entity. From this notion of birth, the developing entity in an AW is not “completely born,” since it conserves fetal characteristics [14]. Considering that (live) birth is one of the conditions for obtaining strong moral (and legal) status in Canada [15], the developing entity in an AW may not hold these statuses if it is not considered completely born.

By contrast, Colgrove considers the developing entity in an AW to be a newborn, since it complies with several internationally accepted definitions of live birth, including those of the World Health Organization (WHO), the European Union, US law, and the international medical community [16]. As an example, Colgrove puts forward the WHO's definition of live birth, which is as follows: “the complete expulsion or extraction from its mother of a product of conception, irrespective of the duration of the pregnancy, which, after such separation, breathes or shows any other evidence of life—for example, beating of the heart, pulsation of the umbilical cord or definite movement of voluntary muscles—whether or not the umbilical cord has been cut or the placenta is attached” [16, p. 724]. Given that newborns are morally considered to be persons in general, Colgrove argues that the developing entity in an AW should be treated as such. However, according to the WHO definition of live birth, a hypothetical 6‐week‐old embryo extracted from a human uterus and transferred to an AW would be considered born, since it would be completely extracted from its mother and would show signs of life. Indeed, an embryo's heartbeat is detectable by Doppler ultrasound from the 6th week after conception [17]. Considering a developing entity in an AW to have been born at such an early stage of development involves major ethical and legal issues. Colgrove himself acknowledges the possible need to modify the definition of “live birth,” mentioning, however, that this question is not addressed in his essay, concentrating only on demonstrating that the developing entity in an AW satisfies current definitions of “live birth” [18]. Romanis [14], and Kingma and Finn [11] believe that redefining the concept of “live birth” to consider the transition from fetal to neonatal characteristics would avoid recognizing an entity as having been born alive at such an early developmental stage.

However, Wozniak presents three case studies supporting the view that fetal characteristics can be preserved following environmental birth, that is, after having exited the gestational body, but without being transferred to an AW [19]. Thus, Romanis' as well as Kingma and Finn's vision would imply that many newborns currently recognized as persons would have this status withdrawn, since they retain some of their fetal characteristics. However, Kingma and Finn were already concerned that their redefinition of birth could lead to certain gray areas. This is why they argued that technologies allowing fetal and neonatal characteristics to be present simultaneously would not pose a problem to the position that they advocate for. It would be sufficient to determine which characteristics should surpass the others to determine whether the developing entity in an AW is a newborn or not [11]. If this proposal is accepted, further work would need to be carried out to determine the characteristics required to be considered a newborn. It is beyond the scope of this article to determine by whom and on the basis of what standards these reflections should be carried out, but special care must be taken to ensure that the most vulnerable patients are not left in an ethical and legal limbo.

## 3 The Fetus as a Patient's (TFAAP) Proposal by Chervenak and Mccullough

In this section, TFAAP's proposal will be presented in its original version, before being transposed to the context of partial ectogenesis in the following section. TFAAP's proposal has emerged in a context where screening tests and treatments are increasingly available for the fetus in a human uterus via the body of the person carrying it [12]. Given this context, the authors proposed TFAAP's framework to guide decision‐making within an obstetrician–patient relationship [20].

According to these authors, it is not necessary to have independent moral status to be considered and treated as a patient. Independent moral status for the fetus means “that one or more characteristics that the fetus possesses in and of itself and, therefore, independently of the pregnant woman or any other factor, generate and therefore ground obligations to the fetus on the part of the pregnant woman and her physician” [12, p. 401].

Although numerous moments (fertilization, implantation, quickening, etc.) have been suggested for the acquisition of an independent moral status by the fetus, no consensus on the matter has emerged as yet. For this reason, Chervenak and McCullough abandon the idea of linking the concept of “fetus as a patient” with independent moral status.

Thus, the fetus, even if it does not have independent moral status, can be considered a patient under two conditions established by Chervenak and McCullough. The first condition is that the pregnant person chooses to present their fetus to physicians so the latter can carry out a medical procedure. The second condition is the existence of medical interventions that are beneficial to the fetus. The authors state that if these two conditions are met, the fetus has a dependent moral status (i.e., moral agents have moral obligations toward it, even if the conditions that must be met are not solely related to intrinsic characteristics of the fetus). However, they add that the fetus‐patient should have a reasonable potential to acquire an independent moral status for physicians to have moral obligations toward it. Chervenak and McCullough consider that the acquisition of independent moral status is closely linked to the concept of viability, as they state: “a viable fetus can exist ex utero and thus achieve independent moral status” [12, p. 402].

Considering that any medical intervention on the fetus in a human uterus will necessarily pass through the body of the pregnant person, the latter must consent to present their fetus to physicians; otherwise, their fetus cannot be considered a patient. For this reason, physicians have obligations relating to the respect for the autonomy of pregnant patients. Obligations to respect the patient's autonomy require that reasonable alternatives (diagnoses and treatments) are presented to the patient. Once patients have understood this information, they are in a position to evaluate their options and establish their preferences. Physicians must then take the patient's preferences and beliefs into account (unless there is a legitimate reason not to) [12]. This concretely means that whether to undergo the fetal treatment is ultimately a decision of the pregnant person, who is entitled to refuse the treatment regardless of fetal benefit and of the partner's opinion [21]. As the fetus‐patient would not be able to demonstrate preferences and beliefs due to its underdeveloped central nervous system, physicians have no moral obligation regarding fetal autonomy [12].

Considering that physicians have an obligation to protect and promote the interests of patients in terms of health, the former have beneficence‐based obligations toward the pregnant person and the fetus‐patient. Once the fetus is a patient, it would be ethically justified to recommend medical management in accordance with the principle of beneficence [12]. The authors specify that in medical ethics, “beneficence‐based obligations are a direct function of evidence‐based clinical judgment about diagnostic and therapeutic measures that are reliably expected to result in a greater balance of clinical goods over clinical harms for patients” [12, p. 3]. However, recognizing the fetus in the human uterus as a patient could cause conflicts of interest between the pregnant patient and the fetus‐patient [22]. In so doing, the rights of the pregnant person could be diminished if physicians prioritize the interests of the patient‐fetus over those of the pregnant person.

Nonetheless, physicians are also adamant that beneficence obligations toward the fetus “must, in all cases, be considered along with beneficence‐based and autonomy‐based obligations to the pregnant woman” [21, p. 11]. Thus, TFAAP's proposal warns that the rights of the pregnant person are diminished by the fact that their fetus is considered a patient; although physicians are allowed to make recommendations based on fetal benefit, the pregnant person is the only one entitled to make a final decision. Potential conflicts of interest are minimized even further in the context of partial ectogenesis as the developing entity in an AW is no longer inside the human body of a third party. While it is beyond the scope of this article to state on whether the fetus in the human uterus should be considered a patient, we do argue that the entity in an AW should be considered as such, considering the factor of exteriority to the human body.

## 4 The Proposition “The Fetus as a Patient” Transposed to the Developing Entity in an AW

The aim of this section is to analyze TFAAP's proposal in the context of partial ectogenesis. According to this proposal, the developing entity in an AW would be a patient, since it meets the two conditions established by the authors. For the first condition, the pregnant person will have presented their fetus (future developing entity in an AW) to physicians for extraction from the human uterus and subsequent transfer to the AW. As for the second condition, once the AW has demonstrated satisfactory proof of its efficacy, it would be presented as a beneficial medical intervention for the fetus/entity in an AW. Moreover, viability, in relation to ex utero existence, as put forward by Chervenak and McCullough, joins several accepted definitions of this first concept. For example, the *Merriam Webster Dictionary* and the *American Heritage Dictionary* define respectively viability as “the capability of a fetus to survive outside the uterus”^4^ and “Capable of living outside the uterus. Used of a fetus or newborn.”^5^ For these reasons, it appears that the developing entity in an AW should minimally hold a dependent moral status. Therefore, physicians would have beneficence‐based obligations toward it.

It should be remembered that the pregnant person is under no obligation to present their fetus to physicians, even if favorable medical interventions would be available for the fetus. In the context of partial ectogenesis, this would mean that the pregnant person has the right to refuse to transfer their fetus to the AW, considering that this medical procedure will require a Cesarean section, which includes a risk of complications [23]. Thus, forcing the transfer against the pregnant person's would be considered as a violation of their rights to autonomy [24]. However, it is interesting to note that once the pregnant person has presented their viable fetus to physicians, beneficence‐based obligations toward the fetus‐patient can no longer be halted [25]. This would mean that the obligations of beneficence toward the developing entity would have to be maintained once it is in an AW. It would be the case even if the person who previously presented it to the physicians changes their mind and requests an intervention that is not beneficial for the developing entity in an AW. Chervenak and McCullough state that “[t]he benefits that medicine is competent to seek for patients are the prevention and management of disease, injury, handicap, unnecessary pain, and suffering, and the prevention of premature or unnecessary death” [12, p. 400].

To illustrate concretely how considering the developing entity in an AW as a patient would translate into practice, we analyze the case of termination of an entity's existence requested by one or both (intent) parents.

As described above, considering that the developing entity in an AW would be a patient, the physicians would have the obligation to protect and promote its health interests. Even if it is reasonable to assume that the developing entity in an AW cannot demonstrate conscious, rational interests, we argue that physicians must still take into account its current interests (e.g., avoiding unnecessary pain) and future interests (e.g., ensuring a better quality of life in terms of health). Thus, the developing entity in an AW would be treated according to neonatal/pediatric care standards because of its patient status, ensuring that its future interests would be considered despite its lack of rationality, as is currently the case for young pediatric patients. As the future health interests of the developing entity in an AW should be respected by virtue of its patient status, it would be protected from unnecessary death (i.e., death that is not in its best interests). However, we prefer the concept of “good enough interests” to that of best interest, considering that it is difficult, if not impossible, to achieve the latter at all times and on all levels [26]. Legitimate decisions within the former's approach range from “those that are in the absolute best interests of the child, to those that partially meet certain interests without causing considerable harm to the child” [Free transl.] [26, p. 265]. By replacing the term “child” in this statement with “developing entity,” we conclude that terminating the existence of the latter would not always be legitimate, as it could be a considerable harm to the developing entity in an AW. This would be the case if the existence of the entity in an AW were terminated without the presence of health conditions that would significantly compromise its current or future quality of life. Thus, medicine would fail to avoid premature or unnecessary death, and consequently, would not respect the (future) good enough interests of the developing entity in an AW.

In Canada, considering that abortion is decriminalized throughout the whole gestation, regardless of the reasons behind this choice [27], the good enough interests of a potential child are not necessarily considered. In doing so, Canadian abortion standards should not apply to the developing entity in an AW, since this would not respect the beneficence‐based obligations that physicians have toward their patients. Therefore, we argue that if the end of the developing entity's existence in an AW is requested, only a cessation of treatment (withdrawing life‐sustaining treatment), followed by palliative care, would be ethically acceptable. Indeed, this medical intervention (like any others) must respect the patient's interests but “is commonly thought to be justified when […] death is imminent and continued treatment is judged to be futile (or excessively burdensome)” [28].

The example of terminating the existence of the developing entity in an AW demonstrates why it is relevant to consider the latter as a patient. The moral, ethical, and legal guidelines that physicians must follow in their practice inform how the existence of the developing entity in an AW should be terminated (if applicable). Following the good enough interests’ approach, the developing entity in an AW would not only be treated according to the principle of beneficence but also according to the principle of non‐maleficence, consisting of avoiding causing harms [12]. These moral obligations are also reinforced within jurisdictions, such as in the Canadian Medical Association's Code of Ethics and Professionalism, which stipulates: “Consider first the well‐being of the patient […] Take all reasonable steps to prevent or minimize harm to the patient […] act to bring about a positive balance of benefits over harms” [29].

Considering the developing entity in an AW as a patient also has the advantage of including all (future) holders of parental authority in medical decisions concerning the former. In this way, Canadian standards of consent to care for a minor patient would apply, with decision‐making power shared between the (future) parents or guardians. On the other hand, if the developing entity in an AW was not considered to be a patient, it could possibly be treated according to current Canadian abortion standards. In *Tremblay v. Daigle*, the Supreme Court of Canada ruled that only the pregnant person has the right to decide whether to have an abortion, leaving no decision‐making power to the other potential parent [15]. While we do not question this decision in the case of human gestation, we believe that it would be unjustified to grant all decision‐making power to the person who previously carried the fetus transferred to an AW. We take this position because when the fetus is transferred to the AW, *human gestation* ends, and with it also ends any threat to the bodily integrity of a third party.

As a result, TFAAP's proposal would ensure shared decision‐making and protect the developing entity in an AW from certain medical and parental harms, even if it does not have independent moral status. Thus, the relevance of TFAAP's proposal lies in the fact that it generates guidelines for ethically treating the developing entity in an AW, while bypassing the thorny question of its nature (fetus, newborn, or new entity). Importantly, TFAAP's proposal ensures that beneficial treatment is provided, while avoiding the risk of therapeutic obstinacy, as it is still possible for parents and healthcare providers to withhold or withdraw AW if they deem it against the good enough interests of the developing entity.

## 5 Conclusion

To conclude, this article sought to determine how the developing entity in an AW should be ethically considered. Following our analysis of the TFAAP's proposal by Chervenak and McCullough, transposed to the context of partial ectogenesis, we have seen that the developing entity in an AW meets the conditions established by the authors to be considered as a patient. Indeed, the developing entity in an AW would be presented to physicians, could benefit from medical treatment, and would have the potential to obtain independent moral status. With these conditions met, the developing entity in an AW would be a patient. This status should result in the patient being treated according to the principle of beneficence and would therefore avoid unnecessary suffering or unwarranted premature death. For these reasons, we argued that the developing entity in an AW should be considered as a patient.

Furthermore, the thesis that we defend fills certain gaps in the theories that attempt to determine the moral status of the developing entity in an AW. We have seen that questions concerning the classification of AW technology and the concept of birth as applied to the developing entity in an AW fail to clearly determine the latter's ethical treatment. Considering these shortcomings, TFAAP's proposal transposed to the context of partial ectogenesis emerged as the most ethically acceptable.

We maintain that TFAAP's proposal remains relevant to the treatment of the developing entity in an AW, since it does not need to have independent moral status to be considered a patient. Thus, even if some people consider the developing entity in an AW to be more like a fetus than a newborn, it would still be protected from certain parental and medical harms by virtue of its patient status. As physicians have obligations of non‐maleficence and beneficence toward their patients, the developing entity in an AW will have to be treated according to the principle of good enough interests, including a zone of parental discretion. This approach implies that good enough parenting decisions should be tolerated, even if they are not optimal, unless they cause obvious harm to the child [30] (or in our case, to the developing entity in an AW). Therefore, considering the developing entity in an AW as a patient achieves a balance between the principles of non‐maleficence, beneficence, and autonomy, which seems to be the best ethical compromise for the developing entity in an AW, (expecting) parents, and healthcare providers.

## Data Availability

Data sharing is not applicable to this article as no data sets were generated or analyzed during the current study.

## Endnotes

^1^
Several appellations have been used to name the developing entity in an AW, including fetus, newborn, gestateling, fetonate, fetal neonate, ectogenetic fetus, and so on. The term ‘developing entity in an AW’ seems to us to be more appropriate because of its neutrality, thus avoiding inducing moral and legal implications in an entity for which its moral and legal status remains undetermined.

^2^
There is some debate about the terminology used to describe the AW‐supported process. Some authors prefer to use the term “ectogestation” rather than “ectogenesis,” each reflecting a different school of thought. We have chosen to use only the term “ectogenesis” for simplicity's sake, without taking a position on which school of thought should be prioritized.

^3^
Although defining the legal status of the developing entity in an AW is clearly important, this is beyond our expertise of ethicists and philosophers, Further, the legal status has already been thoroughly debated by jurists.

^4^
Merriam Webster Dictionary. Viability.

^5^
American Heritage Dictionary. Viability.
